# Dynamic Die-Forging Scene Semantic Segmentation via Point Cloud–BEV Feature Fusion with Star Encoding

**DOI:** 10.3390/s26020708

**Published:** 2026-01-21

**Authors:** Xuewen Feng, Aiming Wang, Guoying Meng, Yiyang Xu, Jie Yang, Xiaohan Cheng, Yijin Xiong, Juntao Wang

**Affiliations:** 1 School of Mechanical and Electrical Engineering, China University of Mining and Technology-Beijing, Beijing 100083, China; 2 School of Vehicle and Mobility, Tsinghua University, Beijing 100084, China; 3 Beijing China Coal Mine Engineering Co., Ltd., Beijing 100013, China; 4 State Key Laboratory of Digital Intelligent Technology for Unmanned Coal Mining, Zhangjiakou 076250, China; 5 China Coal Zhangjiakou Coal Mining Machinery Co., Ltd., Zhangjiakou 076250, China; 6 Hebei Province High-end Intelligent Mine Equipment Technology Innovation Center, Zhangjiakou 076250, China

**Keywords:** point cloud, forging, star-operation, alignment mechanism, feature fusion

## Abstract

Semantic segmentation of workpieces and die cavities is critical for intelligent process monitoring and quality control in hammer die-forging. However, the field of 3D point cloud segmentation currently faces prominent limitations in forging scenario adaptation: existing state-of-the-art (SOTA) methods are predominantly optimized for road driving or indoor scenes, where targets have stable poses and regular surfaces. They lack dedicated designs for capturing the fine-grained deformation characteristics of forging workpieces and alleviating multi-scale feature misalignment caused by large pose variations—key pain points in forging segmentation. Consequently, these methods fail to balance segmentation accuracy and real-time efficiency required for practical forging applications. To address this gap, this paper proposes a novel semantic segmentation framework fusing 3D point cloud and bird’s-eye-view (BEV) representations for complex die-forging scenes. Specifically, a Star-based encoding module is designed in the BEV encoding stage to enhance capture of fine-grained workpiece deformation characteristics. A hierarchical feature-offset alignment mechanism is developed in decoding to alleviate multi-scale spatial and semantic misalignment, facilitating efficient cross-layer fusion. Additionally, a weighted adaptive fusion module enables complementary information interaction between point cloud and BEV modalities to improve precision.We evaluate the proposed method on our self-constructed simulated and real die-forging point cloud datasets. The results show that when trained solely on simulated data and tested directly in real-world scenarios, our method achieves an mIoU that surpasses RPVNet by 1.1%. After fine-tuning with a small amount of real data, the mIoU further improves by 5%, reaching optimal performance.

## 1. Introduction

During the hammer forging process, a workpiece is subjected to frequent pose variations and severe surface deformation under high temperature and pressure conditions. Such non-rigid deformations significantly increase the difficulty of 3D point cloud-based object recognition. While recent point cloud recognition techniques, such as PointNet [1] and PointNet++ [2], have made considerable progress in semantic understanding, they are primarily designed for environments with relatively stable geometric structures (e.g., roads and indoor scenes). Consequently, these methods are ill-suited for recognizing complex, non-rigid workpieces in industrial manufacturing settings.

Based on different representation paradigms, current 3D point cloud semantic segmentation methods can be broadly categorised into two groups: 3D representation-based methods and 2D projection-based methods. 3D representation-based approaches primarily include methods that process raw point clouds directly and those that utilise voxel representations. Raw point cloud-based methods, such as DGCNN [3] and RandLA-Net [4], typically employ specialised modules to extract features directly from the unordered point sets. DGCNN [3] constructs graph structures to capture local geometric relationships within the point cloud, while RandLA-Net [4] employs an efficient local feature aggregation scheme to handle large-scale point cloud data. Voxel-based methods, exemplified by SparseConv [5] and Cylinder3D [6], first convert point clouds into structured voxel grids and then utilise 3D convolutional operations for feature extraction, enabling effective neighbourhood feature learning and object recognition. SparseConv [5] enhances computational efficiency through sparse convolution, whereas Cylinder3D introduces a cylindrical voxel partitioning strategy to improve long-range contextual modelling. While these 3D representation-based methods effectively preserve spatial geometric information, they often require substantial computational resources during both training and inference, leading to slower processing speeds.

A 2D-projection-based method for point-cloud semantic segmentation first projects the point cloud onto a 2D plane; then, 2D convolutional operations are used to extract planar features and perform semantic understanding on the 2D plane; finally, the 2D semantic features are back-projected into 3D space to achieve semantic understanding of the 3D point cloud. The 2D planar projection can be implemented either as a bird’s-eye view (BEV) or a range view (RV). For example, RangeNet++ [7] converts the point cloud into a range image via spherical projection and applies a 2D convolutional neural network for semantic segmentation, achieving good performance. PolarNet [8] adopts a projection in polar coordinates on the BEV and uses convolutional neural networks to perform point-cloud semantic segmentation, yielding excellent results. Projection-based point-cloud semantic segmentation methods offer computational efficiency advantages, but they may suffer from partial loss of spatial information.

To harness the complementary advantages of different point cloud representations, recent research has introduced multi-view fusion strategies, which primarily fall into two categories: (1) the fusion of range view and bird’s-eye view representations, where methods like AMVNet [9] integrate BEV and RV features at late stages to mitigate the limitations of individual projection perspectives; (2) the fusion of 3D point clouds with 2D projected views, exemplified by RPVNet [10] that combines raw point cloud, voxel, and range view features during decoding, and CPGNet [11] which employs a joint architecture with dedicated point cloud, RV, and BEV branches to effectively enhance per-point semantic segmentation performance.

Inspired by multi-view fusion frameworks, we introduce a dual-branch semantic segmentation network specifically designed for forging point clouds, which simultaneously exploits raw 3D geometry and structured BEV representations. One branch processes point clouds directly to preserve detailed spatial information, while the other operates in the BEV domain to efficiently encode contextual features. In the BEV encoder, a star-based encoding module is adopted to improve feature expressiveness, and a hierarchical alignment strategy in the decoder ensures robust spatial consistency across scales. To facilitate effective cross-view interaction, we further incorporate a weighted adaptive fusion module that dynamically balances contributions from both views, resulting in superior segmentation accuracy and robustness over conventional single-view models.

In summary, the main contributions of this work are threefold:

1. We construct a comprehensive semantic dataset of forging point clouds encompassing both simulated and real-world scenarios, providing an essential data foundation for 3D recognition tasks in complex industrial environments.
2. We propose a novel semantic segmentation model that integrates 3D point clouds with Bird’s-Eye View (BEV) for forging applications. Specifically, for the BEV branch encoding stage, we design a star-based encoding module and a hierarchical feature alignment mechanism to enhance the encoder’s nonlinear mapping capability and feature representation capacity. During the BEV decoding phase, we introduce a multi-level feature offset calibration module to address feature misalignment caused by downsampling operations, enabling effective feature alignment throughout the upsampling process. Furthermore, we develop a weighted adaptive feature fusion module to achieve dynamic integration of cross-view features between the point view and BEV representation, significantly improving the accuracy and robustness of forging point cloud segmentation.
3. The results obtained by training on the synthetic dataset and testing on the real dataset show that PBNet improves the mIoU metric by 1.1% compared to RPVNet. After further fine-tuning on the real dataset, PBNet achieves an mIoU of 85.9%, still significantly outperforming RPVNet and PTv3 and maintaining the best performance.

## 2. Related Work

Point-based Segmentation. PointNet [1] pioneered direct processing of point clouds by using shared MLPs to extract per-point features and symmetric functions to aggregate unordered point sets, establishing an effective paradigm for point cloud segmentation. While subsequent refinements [2,12,13,14,15] have demonstrated strong performance on indoor benchmarks, most remain constrained by computational and memory limitations when scaling to large-scale scenarios. To address these challenges, RandLA-Net [4] introduced random sampling with local feature aggregation to minimize information loss, while KPConv [15] achieved notable accuracy through its innovative kernel point convolution. Most recently, Point Transformer V3 [16] has advanced the field by specifically targeting the accuracy-efficiency trade-off in point cloud processing. Despite generally maintaining low parameter counts, point-based methods remain constrained by their notoriously inefficient local neighborhood queries, which lead to substantial computational overhead during feature extraction.

Voxel-based Segmentation. Voxel-based methods [17,18,19,20,21,22,23] typically convert unstructured point clouds into regular voxel grids and employ 3D convolutional networks for semantic segmentation. Since standard 3D convolutions incur substantial computational and memory costs in high-resolution scenarios, researchers have developed various improvements to alleviate this spatiotemporal complexity. In 2019, Choy et al. proposed Minkowski Convolutional Neural Networks [24], which significantly reduce the computational burden of 3D feature extraction through sparse tensor representations. In 2020, Tang et al. further introduced Sparse Point-Voxel CNNs [5], effectively reducing computational complexity while maintaining representational capacity. That same year, Zhu et al. developed Cylinder3D [6], which utilises asymmetric residual blocks to minimise redundant computations while better preserving geometric features of elongated objects. Building upon these advances, AF2S3Net [25] incorporates an Attention Feature Fusion Module (AF2M) and an Adaptive Feature Selection Module (AFSM) to more effectively integrate local and global contexts while emphasising fine-grained detail features. Nevertheless, voxel-based methods still face significant information loss at low resolutions, while high resolutions lead to dramatically increased computational complexity, creating an inherent and unavoidable trade-off between accuracy and efficiency in voxel-based modelling.

2D Projection-based Segmentation. Compared to point-based and voxel-based approaches, projection-based methods generally achieve faster inference speeds. The core idea involves projecting sparse 3D point clouds onto regular 2D representation spaces, thereby leveraging well-established 2D convolutional or Transformer architectures for feature extraction. These methods are mainly categorised into two types: Range View (RV) projection and Bird’s-Eye View (BEV) projection. In RV-based methods, RangeNet++ [7] was the first to systematically explore the potential of mapping point clouds into range images combined with 2D convolutions. It also introduced a GPU-accelerated k-nearest neighbours (KNN) post-processing scheme to ensure semantic consistency within local point neighbourhoods. Subsequently, RangeViT [26] incorporated pre-trained Vision Transformers (ViT) along with a point-wise semantic refinement module, significantly improving semantic prediction accuracy. Building upon this foundation, RangeFormer [27] established an end-to-end RV framework that integrated a novel network architecture, data augmentation techniques, and post-processing mechanisms. It further introduced a Scalable Training from Range View (STR) strategy, substantially enhancing the applicability and generalisation capability of RV-based methods. In contrast, BEV-based methods focus more on modelling global spatial structures in the bird’s-eye plane. PolarNet [8] pioneered the use of polar coordinate BEV representations to mitigate the non-uniform point distribution issues inherent in Cartesian coordinates and designed ring-shaped convolutions to adapt to the polar coordinate space. Following this direction, Panoptic-PolarNet [28] extended semantic segmentation by incorporating instance clustering, achieving panoramic LiDAR point cloud segmentation and demonstrating stronger scene understanding capabilities.

Multi-View Fusion. Given the inherent limitations of single-view representations in expressive power, recent years have witnessed the emergence of various methods [9,29,30,31,32] that attempt to fuse features from multiple views. For instance, refs. [29,30] integrate point-level information from both Bird’s-Eye View (BEV) and Range View (RV) at the input stage, achieving early fusion of cross-view features. In contrast, AMVNet [9] proposes an uncertainty-aware late fusion strategy, which estimates the uncertainty of predictions from different views and employs an additional network to refine the results, thereby enhancing segmentation consistency. FusionNet [31] designs a point-voxel interactive MLP that efficiently aggregates features between neighbouring voxels and corresponding points, reducing the computational overhead of neighbourhood search while maintaining a favourable balance between accuracy and efficiency on large-scale point cloud data. PVCNN [33] offers a lightweight point-voxel fusion scheme, where the voxel branch provides coarse-grained local structural information while the point branch preserves fine-grained geometric features through point-wise MLPs. RPVNet [10] integrates three distinct representations—points, voxels, and range views—constructing a deep fusion framework with multi-modal interactive information flow and designing a gated fusion module to adaptively combine features from the three branches. Furthermore, CPGNet [11] combines BEV, RV, and raw point clouds to propose a multi-modal fusion model that achieves an improved balance between accuracy and inference speed. Overall, these multi-view fusion methods effectively mitigate the shortcomings of single-view representations by leveraging complementary advantages, demonstrating enhanced robustness and generalisation capability in complex scenarios.

## 3. Data and Methods

This section first briefly introduces the data acquisition equipment and sample data, then outlines the overall architecture of the proposed model, and provides a detailed description of its core components: the bidirectional projection mechanism between point clouds and Bird’s Eye View (BEV), the star-operation feature encoding strategy and the multi-level feature alignment module in the BEV branch, and the final weighted feature fusion module.

### 3.1. Data Acquisition

To address the failure of traditional visual recognition methods under harsh forging conditions such as intense light and high temperatures, this study employs a Mech-Eye LSR L long-range industrial 3D laser scanner to collect point cloud data on both a high-fidelity simulated forging platform and a real industrial production line. The Mech-Eye LSR L is an industrial-grade 3D vision sensor capable of generating high-quality, detailed 3D point clouds with strong resistance to ambient light interference, a large field of view, and precise geometric measurements even under challenging lighting conditions. It is particularly suitable for factory automation and robotics guidance tasks in environments with strong environmental interference, such as bright reflections and dynamic lighting changes.

**Figure 1:**
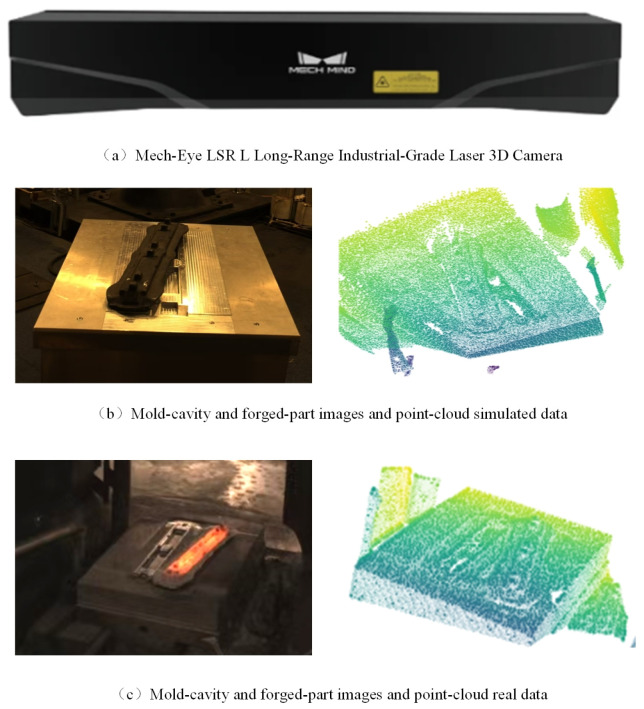
Data acquisition device and collected samples. (**a**) Diagram of the Mech-Eye LSR L long-range industrial 3D laser camera; (**b**) Image and point cloud data acquired in the simulated environment; (**c**) Image and point cloud data obtained from the real-world forging scenario. Comparative analysis between subfigures (**b**,**c**) reveals substantial differences in the image data across different environments, while the corresponding forging point clouds maintain high structural consistency.

As illustrated in Figure 1, during the hammer forging process, 2D images are highly susceptible to environmental disturbances (e.g., drastic illumination variations, thermal radiation, and specular reflections), which can lead to significant distortion or instability of appearance information. This inconsistency greatly increases the difficulty of transferring image-based semantic understanding methods from simulation to reality, thereby limiting their applicability in real production scenarios. In contrast, 3D point clouds exhibit excellent geometric consistency across different scenes: the global topology and local geometric details of the workpiece and die cavity remain highly stable in both simulated and real forging environments. Such robustness makes 3D point clouds an ideal input modality for modeling and recognition tasks, effectively mitigating the challenges caused by appearance changes due to environmental interference.

Based on the above observations, we select point clouds as the core data source for semantic segmentation of die cavities and forged workpieces. By leveraging the structural consistency of point clouds in both simulation and real-world scenarios, we significantly reduce the domain gap between simulated training data and real test data, providing a feasible path toward cross-domain generalization without requiring extensive real-world annotations.

### 3.2. Framework Overview

**Figure 2:**
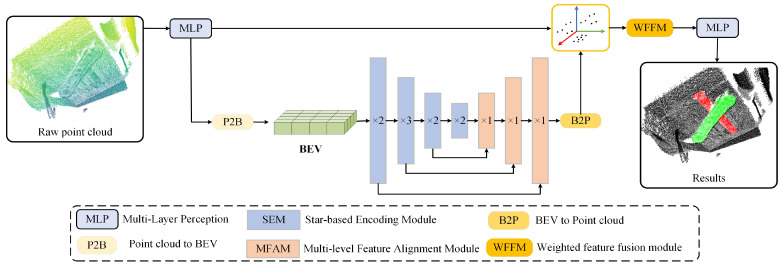
Network architecture of the point cloud and Bird’s-Eye View feature fusion model (PBNet). The model consists of an original point cloud branch and a bird’s-eye-view (BEV) branch. “×L” denotes the number of repeated layers in each component. The BEV features are constructed via P2B projection. BEV features are then projected back into the 3D space through a B2P operator.Arrows indicate the feature propagation and fusion process, while different colors are used to distinguish different functional modules.

The overall architecture of the proposed PBNet is illustrated in Figure 2. The framework consists of two main components: a raw point cloud branch and a Bird’s-Eye View (BEV) branch. The raw point branch employs a two-layer Multi-Layer Perceptron (MLP) for initial per-point feature extraction, followed by a point-to-BEV (P2B) projection that maps the point features onto the BEV plane to efficiently construct neighbourhood representations in the 2D domain. The encoder of the BEV branch comprises four stages, each containing multiple stacked star-based encoding modules (SEM) designed to progressively enhance the representation capacity of local features and improve spatial context modelling. In the decoding phase, a multi-level feature alignment module(MFAM) is introduced to alleviate potential spatial misalignment and feature distortion caused by downsampling, ensuring effective integration of multi-level features. Following BEV feature extraction, a BEV-to-point (B2P) operation is applied to back-project the BEV features into 3D space, where they are adaptively fused with the original point features via a weighted feature fusion module (WFFM), yielding semantically enriched output representations. Finally, the fused features are mapped to predefined point-wise categories, achieving high-precision semantic segmentation of forged workpieces.

### 3.3. Point-to-BEV and BEV-to-Point

**Figure 3:**
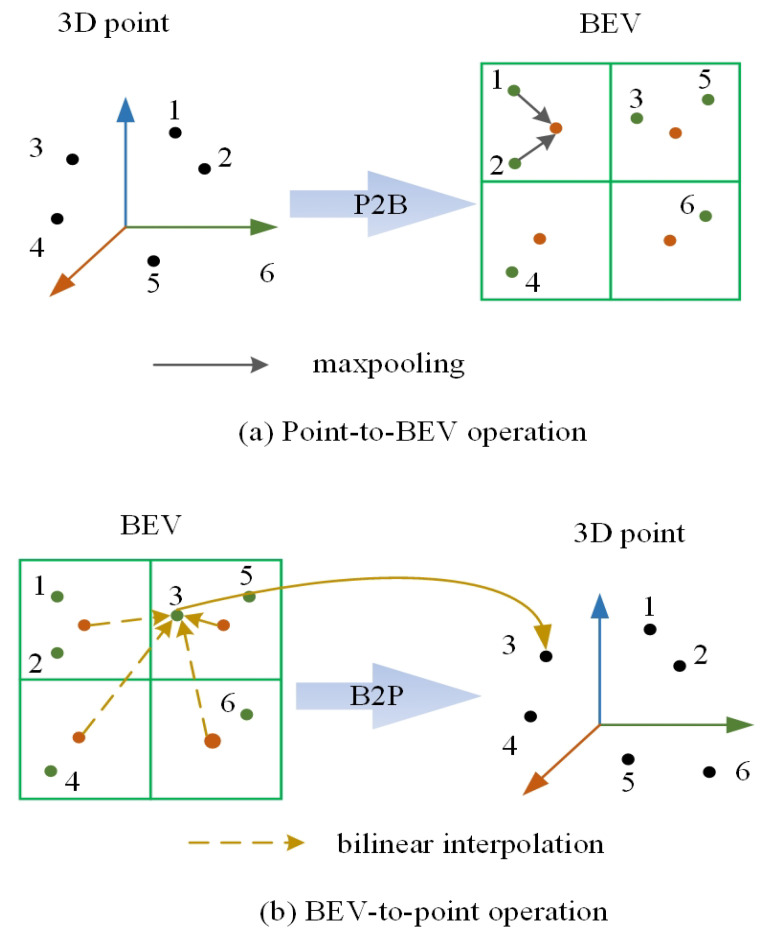
Diagram of (**a**) P2B and (**b**) B2P operations.

The Point-to-BEV (P2B) operation [11] is illustrated in Figure 3a, where the black arrows represent max-pooling operations used to aggregate 3D spatial points projected into the same grid cell, thereby obtaining 2D grid coordinates corresponding to the 3D coordinates. The Point-to-BEV projection is formally described as follows: Given a point cloud set $P=\{p_{j}|j=1,\ldots ,N\}$, where *N* represents the total number of points in P, we take an arbitrary point $p_{j}$ with 3D coordinates $\left(x_{j},y_{j},z_{j}\right)$ and project it onto the Bird’s-Eye View (BEV) plane, as illustrated by the green point in Figure 3a, to obtain the corresponding 2D coordinates $\left(u_{j},v_{j}\right)$. During this projection, multiple points from the point cloud set P can be projected onto the same 2D grid cell. Therefore, let the set $G_{h,w}$ denote the indices of all points $p_{j}$ that fall into the same grid cell (h,w), as shown by the orange points in Figure 3a. Formally, this can be expressed as:

(1) $$\begin{array}{c} G_{h,w}=\left\{j|h=\left\lfloor u_{j}\right\rfloor ,w=\left\lfloor v_{j}\right\rfloor \right\}, \end{array}$$

where ⌊·⌋ denotes the floor function. After completing the projection of point features, a max-pooling operation is applied to aggregate the point features $F_{j,c}^{3D}$ within $G_{h,w}$, generating the corresponding BEV feature $F_{h,w,c}^{2D}$. Thus, the operation of projecting 3D points to 2D grid cells is formulated as follows:

(2) $$\begin{array}{c} F_{h,w,c}^{2D}=\underset{j\in G_{h,w}}{max}\left(F_{j,c}^{3D}\right) \end{array}$$

Equation (2) summarizes the point feature aggregation method when projecting 3D point clouds onto a 2D plane, as depicted in Figure 3a. In the bird’s-eye-view (BEV) representation, 3D points are projected onto the x–y plane and discretized via a rectangular 2D grid. The grid covers the boundary range $\left(x_{min},y_{min},x_{max},y_{max}\right)$ and is discretized with a spatial resolution *r* (in meters per pixel), with its width and height are denoted as $W_{bev}=\frac{x_{max}-x_{min}}{r}$ and $H_{bev}=\frac{y_{max}-y_{min}}{r}$, respectively—computed as in Equation (3).

(3) $$\begin{array}{c} \left(\begin{array}{c} u_{j}\\ v_{j} \end{array}\right)=\left(\begin{array}{c} \frac{x_{j}-x_{min}}{x_{max}-x_{min}}\times W_{bev}\\ \frac{y_{j}-y_{min}}{y_{max}-y_{min}}\times H_{bev} \end{array}\right) \end{array}$$

The BEV-to-Point (B2P) operation [11], serving as the inverse of the Point-to-BEV (P2B) projection, aims to remap 2D features back into 3D space. The specific procedure is illustrated in Figure 3b. This operation consists of two main steps: First, the corresponding 2D grid coordinates $\left(u_{j},v_{j}\right)$ are determined for each point, as shown by the orange points in Figure 3b. Then, bilinear interpolation is performed within the four adjacent grid cells surrounding these coordinates. The mathematical formulation is given by:

(4) $$\begin{array}{c} \begin{array}{c} F_{j,c}^{3D}=\sum_{m=0}^{1}\sum_{n=0}^{1}w_{m,n,j}F_{h+m,w+n,c}^{2D}\\ w_{m,n,j}=\left(1-\left|u_{j}-\left(h+m\right)\right|\right)\left(1-\left|v_{j}-\left(w+n\right)\right|\right) \end{array} \end{array}$$

In Equation (4), *h* and *w* represent $\left\lfloor u_{j}\right\rfloor$ and $\left\lfloor v_{j}\right\rfloor$ respectively, where ⌊·⌋ denotes the floor operation. It should be noted that adjacent grid cells falling outside the valid 2D grid boundaries are treated as zero vectors (zero-padding). Importantly, this computation is performed independently for each point and each feature channel, making it particularly suitable for parallel implementation on CUDA-enabled architectures.

### 3.4. BEV Branch Encoding Module

#### 3.4.1. Star-Based Encoding Module

**Figure 4:**
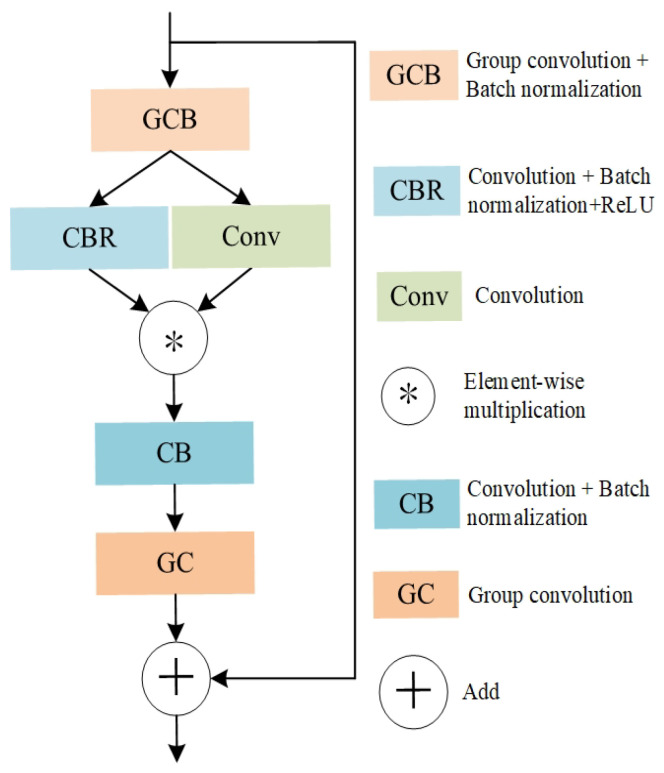
Star-based Encoding Module. This module incorporates grouped convolutional operations and a Star-based operator (*) to effectively capture representative point cloud features, thereby further enhancing encoding efficiency.

The encoder of the BEV branch is built upon a star-based encoding module, as shown in Figure 4. Following the design principle of residual networks, the module introduces a star operation (i.e., element-wise multiplication) to nonlinearly project input features into a high-dimensional representation space, without incurring a significant increase in model complexity.

Concretely, the input features are first processed by a grouped convolution followed by batch normalization (GCB) to enable channel-wise grouping and reduce computational overhead. The resulting features are then split into two parallel branches: one branch applies a convolution, batch normalization, and ReLU (CBR) block to enhance nonlinearity, while the other branch employs a standard convolution to capture local features. The outputs of the two branches are fused via a star operation (*), performing element-wise multiplication to facilitate adaptive feature interaction and modulation in the high-dimensional space.

The modulated features are subsequently refined by a convolution with batch normalization (CB) and a grouped convolution (GC) for feature aggregation and dimensionality reduction. Finally, a residual connection adds the original input to the output of the module, which effectively mitigates vanishing gradients and stabilizes training. Notably, the star operation implicitly corresponds to a mapping into an infinite-dimensional space, as theoretically demonstrated by Xu et al. [34]; further analysis is provided in the Appendix A.

#### 3.4.2. Dual-Branch Subsampling Module

**Figure 5:**
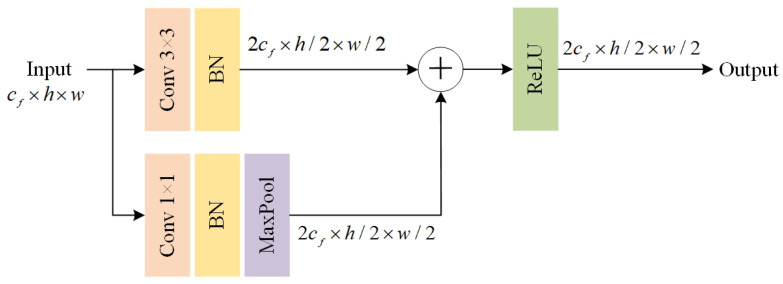
Dual-branch downsampling module. Here, $c_{f}$, *h*, and *w* denote the number of channels, height, and width of the 2D feature map, respectively. BN denotes Batch Normalization.Different colors represent different feature branches, and the “+” symbol denotes feature fusion by element-wise addition.

To more comprehensively preserve critical detail information during the image downsampling process, this paper designs a Dual-branch Downsampling Module (DDM), as illustrated in Figure 5. Conventional single-path downsampling methods often suffer from local detail loss due to information compression when reducing the resolution of feature maps. To address this issue, the DDM employs two parallel, functionally complementary paths working cooperatively: Branch I utilizes a 3 × 3 convolutional layer with a stride of 2 to achieve spatial downsampling while retaining relatively rich contextual structure; Branch II first applies a 1 × 1 convolution to extract local fine-grained features and then performs dimensionality reduction via max-pooling to enhance and preserve the most discriminative local responses. Finally, the feature maps output from the two paths are fused along the channel dimension, thereby improving the representational capacity of the features while compressing resolution, and providing a more comprehensive and discriminative feature representation for subsequent semantic segmentation tasks.

### 3.5. Decoder Module

#### 3.5.1. Multi-Level Feature Alignment Module

**Figure 6:**
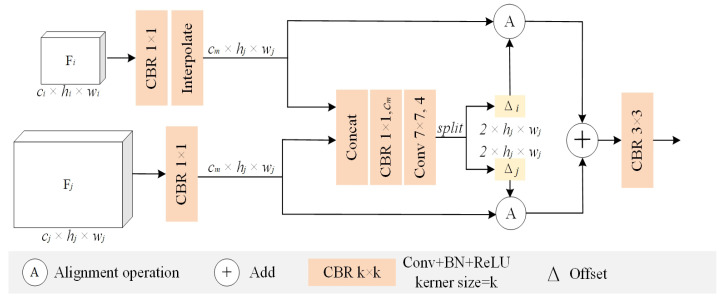
Multi-level feature alignment module.This module takes adjacent decoder-level features as input: a higher-level feature $F_{i}$ with dimensions $c_{i}\times h_{i}\times w_{i}$ and a lower-level feature $F_{j}$ with dimensions $c_{j}\times h_{j}\times w_{j}$, where $c_{i}$ and $c_{j}$ denote the number of channels, and the spatial resolutions satisfy $2h_{i}=h_{j}$ and $2w_{i}=w_{j}$. Here, ${∆}_{i}$ and ${∆}_{j}$ represent the offsets of $F_{i}$ and $F_{j}$, respectively.

The decoder of the BEV branch is composed of a hierarchical feature-alignment module, whose architecture is shown in Figure 6. To effectively preserve edge details, this module first applies convolution and bilinear interpolation to the high-level, low-resolution feature map $F_{i}\in R^{c_{i}\times h_{i}\times w_{i}}$, to unify its channel number and spatial resolution. Then, the upsampled $F_{i}$ is concatenated with the channel-unified $F_{j}\in R^{c_{m}\times h_{j}\times w_{j}}$, and based on this concatenated feature, the module predicts the offsets ${∆}_{i}\in R^{2\times h_{j}\times w_{j}}$ and ${∆}_{j}\in R^{2\times h_{j}\times w_{j}}$ between the upsampled $F_{i}$ and $F_{j}$. The obtained offsets ${∆}_{i}$ and ${∆}_{j}$ are then used to align the high-level feature $F_{i}$ with the low-level feature $F_{j}$, respectively.

Once the offset maps are obtained, the feature alignment and aggregation can be performed according to the following formula:

(5) $$\begin{array}{c} A^{j}=U\left(upsample\left(F_{i}\right),{△}_{i}\right)⊕U\left(F_{j},{△}_{j}\right) \end{array}$$

where $A_{j}$ denotes the aligned aggregated feature, upsample(·) represents the bilinear interpolation function, and U(·,·) indicates the alignment operation. Assume that the spatial coordinates of each position to be aligned on feature map *F* are $\left\{\left(1,1),(1,2),\ldots ,(H,W\right)\right\}$, and the offset map is $∆\in R^{2\times H\times W}$. $U_{hw}$ is the output of the alignment function U(F,∆), which is defined as follows:

(6) $$\begin{array}{c} \begin{array}{c} U_{hw}=\sum_{h^{\prime }=1}^{H}\sum_{w^{\prime }=1}^{W}F_{h^{\prime }w^{\prime }}\cdot max\left(0,1-\left|h+{△}_{hw}^{1}-h^{\prime }\right|\right)\\ \cdot max\left(0,1-\left|w+{△}_{hw}^{2}-w^{\prime }\right|\right) \end{array} \end{array}$$

where the feature value at position $\left(h+{∆}_{hw}^{1},w+{∆}_{hw}^{2}\right)$ on the feature map *F* is obtained via bilinear interpolation. Here, ${∆}_{hw}^{1}$ and ${∆}_{hw}^{2}$ represent the learned 2D spatial offsets corresponding to the position (h,w). It should be noted that if the offsets ∆ are not learned and both ${∆}_{hw}^{1}$ and ${∆}_{hw}^{2}$ in Equation (6) are set to zero, the alignment function will not modify the input feature *F*, and thus the output *U* will be identical to *F*.

#### 3.5.2. Weighted Feature Fusion Module

**Figure 7:**
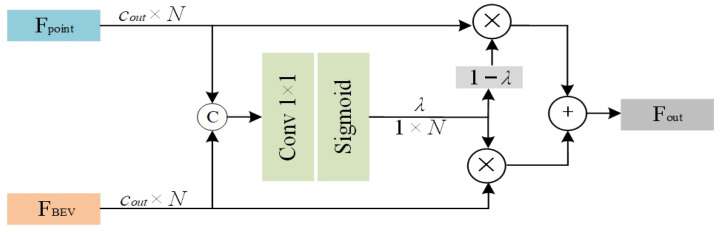
Weighted feature fusion module.The input features consist of the point-view feature $F_{\mathrm{point}}$ and the Bird’s-Eye View feature $F_{\mathrm{BEV}}$. An adaptive weight λ is computed using a sigmoid function at an intermediate stage to perform point-wise feature fusion.Different colors and symbols are used for visual distinction.

After completing BEV feature extraction, the BEV features are projected back into 3D space via the B2P operation. To further integrate point-view and bird’s-eye view features, we design a weighted feature fusion module, as shown in Figure 7. This module adaptively fuses the B2P-projected features $F_{\mathrm{BEV}}$ with the original point-view features $F_{\mathrm{point}}$ to mitigate potential feature loss during the projection process. Specifically, given the point-view features $F_{\mathrm{point}}$ and the bird’s-eye view features $F_{\mathrm{BEV}}$, the weighted fusion module is computed as follows:

(7) $$\begin{array}{c} \begin{array}{c} F_{out}=\lambda F_{BEV}⊕\left(1-\lambda \right)F_{point}\\ \lambda =H\left(F_{point},F_{BEV}\right) \end{array} \end{array}$$

In the Equation (7), $F_{out}$ denotes the output feature, $F_{point}$ represents the original point features, $F_{BEV}$ corresponds to the back-projected Bird’s-Eye View features, λ is the adaptive weight, and H(·) indicates the weight computation function, whose implementation is illustrated in Figure 7.

### 3.6. Loss Function

The segmentation prediction is obtained by applying a fully connected (FC) layer to the output features of the Weighted Feature Fusion Module (WFFM). Due to the pronounced class imbalance in the point cloud semantic segmentation dataset of die cavities and forgings, this work adopts a weighted cross-entropy (WCE) loss function together with the Lovász-Softmax loss to jointly supervise the model training. The overall loss function is defined as follows:

(8) $$\begin{array}{c} L_{total}=L_{CE}+4L_{CE}^{25\%}+3L_{LS} \end{array}$$

(9) $$\begin{array}{c} L_{CE}=-\frac{1}{N}\sum_{n=1}^{N}\sum_{c=1}^{C}y_{n}^{c}log\left({\hat{y}}_{n}^{c}\right) \end{array}$$

(10) $$\begin{array}{c} \begin{array}{c} L_{ls}=\frac{1}{C}\sum_{c=1}^{C}△J_{c}\left(m(c)\right)\\ m_{i}\left(c\right)=\left\{\begin{array}{c} 1-x_{i}\left(c\right),\;if\;c=y_{i}\left(c\right)\\ x_{i}\left(c\right),\;otherwise \end{array}\right. \end{array} \end{array}$$

In Equation (8), $L_{\mathrm{total}}$ denotes the total supervised loss of the network, $L_{\mathrm{CE}}$ is the cross-entropy loss whose explicit form is given in Equation (9) and $L_{ls}$ represents the Lovász-softmax loss, computed as shown in Equation (10). In Equation (9), $y_{c}^{n}$ indicates the semantic ground-truth label, and ${\hat{y}}_{c}^{n}$ is the predicted probability for class *c* at the *n*-th point. To facilitate more accurate classification of hard samples, an additional loss term $L_{CE}^{25\%}$ considers only the top 25% of points with the highest loss values. Furthermore, to maximize the Intersection over Union (IoU) score, the Lovász-softmax loss function proposed by Berman et al. [35] is adopted, which is formulated in Equation (10). In Equation (10), $∆J_{c}$ is defined as the Lovász extension of the Jaccard index, *C* denotes the number of classes, and $x_{i}\left(c\right)\in \left[0,1\right]$ together with $y_{i}\left(c\right)\in \left\{-1,1\right\}$ represent the predicted probability and the ground-truth label, respectively, for class *c* at pixel *i*.

### 3.7. Evaluation Metric

To systematically evaluate the performance of the proposed PBNet and the compared models, this paper adopts the widely used evaluation metric in semantic segmentation the mean Intersection over Union (mIoU) [36]. This metric comprehensively reflects the segmentation accuracy across different categories by calculating the overlap between the predicted regions and the ground-truth annotations for each class and then averaging over all classes. The mIoU is computed as follows:

(11) $$\begin{array}{c} mIoU=\frac{1}{C}\sum_{c=1}^{C}\frac{TP_{c}}{TP_{c}+FP_{c}+FN_{c}} \end{array}$$

where $TP_{c}$, $FP_{c}$, and $FN_{c}$ denote the true positives, false positives, and false negatives for class *c*, respectively, and *C* represents the total number of classes.

## 4. Experiments

The performance of the proposed PBNet is evaluated on both simulated and real-world forge point cloud semantic datasets, demonstrating its effectiveness across different environments.

Simulated Forging Point Cloud Semantic Dataset. To systematically replicate the randomness of workpiece placement and the complexity of working conditions in forging workshops, this study first constructs a simulated forging point cloud semantic dataset. This part is carried out in a controlled laboratory environment, where active introduction of artificial occlusion, multi-angle lighting variations, and background interference simulates extreme observation conditions that may occur in real production lines, such as partial occlusion, glare, and low light. Data collection covers a variety of typical forging shapes and placement poses, ultimately forming a high-quality dataset comprising 350 point cloud frames, with each frame containing approximately 10,000 points. This simulated dataset not only provides diverse training samples for the algorithm but also establishes a controllable and reliable experimental foundation for validating the model’s generalization capability in subsequent real-world scenarios.

Real-scene Forging Point Cloud Semantic Dataset. To further enhance the engineering applicability and scenario coverage of the dataset, the research team conducted on-site point cloud acquisition in an operational forging workshop using a mobile scanning platform. Data were collected during key processes, including heating, forging, and cooling, under actual production conditions. This portion of data originates entirely from real manufacturing environments and incorporates challenges such as complex background interference and deformations caused by high-temperature forging. A total of 350 point cloud frames were captured on-site. Together with the laboratory-simulated data, they form a composite forging point cloud semantic dataset of 700 frames.

In the experiments, the data are partitioned and used as follows. First, to validate the model’s cross-domain generalisation capability, training is conducted on the simulated dataset and evaluation is performed on the real-scene dataset. Second, to further improve the model’s performance in practical scenarios, 350 simulated and 70 real samples are selected and combined to form a training set of 420 samples; the remaining real-scene data is reserved for the final performance evaluation.

### 4.1. Experimental Setup

Network Setup. As shown in Figure 2, the feature extraction modules in PBNet share the same operations but differ in their parameters. In the point branch of PBNet, the MLP at the initial stage takes the 3D coordinates (x,y,z) as input (3 channels) and outputs features with 64 channels.In the BEV branch, the BEV resolution is set to W=640 and H=640. This branch consists of three downsampling and three upsampling stages. The numbers of feature channels in successive stages are 64, 64, 128, 256, 128, 96, and 64, respectively. Consequently, the input to the Weighted Feature Fusion Module (WFFM) are the features from both branches, each with 64 channels, and its output also maintains 64 channels. Finally, point-wise classification is performed through an MLP layer.

Training Details. All experiments were conducted on an NVIDIA RTX 3090 GPU using the PyTorch framework (version 1.12.1) with FP32 precision. The PBNet was trained from random initialisation for 30 epochs with a batch size of 4. Training on two GPUs took approximately 5 h. The optimiser used was Stochastic Gradient Descent (SGD) with a weight decay of 0.001, a momentum of 0.9, and an initial learning rate of 0.02, which was multiplied by 0.1 every 10 epochs.For data augmentation, the following strategies were applied: random rotation around the z-axis, random global scaling sampled from [0.95, 1.05], random flipping along the x-axis and y-axis, and the addition of random Gaussian noise following $N\left(0,0.02\right)$.

### 4.2. Quantitative Results

To validate the domain discrepancy between simulated and real-world data, we first trained our model on the simulated dataset. Then we evaluated it on the real-world forging semantic dataset. The comparative methods include RandLA-Net [4], PTv3 [16], Cylinder3D [6], and the multi-view fusion architectures RPVNet [10] and CPGNet [11], with their core designs described as follows: RandLA-Net adopts a pure point-cloud processing architecture that achieves lightweight computation through random sampling and local feature aggregation; PTv3 (Point Transformer v3) models global geometric relationships in point clouds based on a self-attention mechanism, yielding strong expressive capability; Cylinder3D projects the point cloud into a cylindrical coordinate system and integrates 2D convolution with 3D voxel feature extraction, showing excellent performance in tasks requiring geometric consistency; RPVNet and CPGNet are both multi-view fusion architectures: the former jointly utilizes point-view and voxel features, while the latter fuses point cloud features with BEV semantics, enhancing segmentation accuracy through complementary cross-view information.

As shown in Table 1, under the cross-domain “sim-train/real-test” setting, the proposed PBNet achieves the best performance in semantic segmentation of both forging workpieces and die cavities, with a mean Intersection-over-Union (mIoU) of 80.9%. This result significantly outperforms existing point-based, voxel-based, and multi-view fusion methods, surpassing the previous best model, RPVNet, by 1.1 percentage points. Moreover, PBNet maintains the fastest inference speed (30 ms) while delivering the highest accuracy, substantially outperforming other high-performance models of the same type (e.g., 50 ms for CPGNet and 165 ms for RPVNet). This indicates that our dual-branch fusion architecture of point cloud and Bird’s-Eye View (BEV) can effectively extract and integrate robust cross-view features, thereby largely meeting the real-time processing requirements of industrial scenarios.

To further investigate the model’s adaptability to real-world data, we fine-tuned it using 20% of the real-world forging semantic data. The results are presented in Table 2. After fine-tuning, all compared methods show notable performance improvements, while PBNet still maintains a comprehensive lead: its mIoU rises to 85.9%, which is approximately 5 percentage points higher than the result obtained with only simulated data, and clearly exceeds the current best counterparts, RPVNet (84.8%) and CPGNet (84.2%). In terms of per-category metrics, PBNet reaches 86.6% for forgings and 85.3% for cavities, both being the highest among all methods. Furthermore, PBNet’s inference speed remains consistently fast at 30 ms, far ahead of other models at similar accuracy levels. These results demonstrate that the proposed dual-branch fusion architecture not only possesses excellent cross-domain generalisation ability but can also rapidly adapt to real-world scenarios with only a small amount of real samples. It achieves a superior balance between accuracy and efficiency, providing a reliable solution for industrial real-time point-cloud semantic segmentation.

### 4.3. Qualitative Results

**Figure 8:**
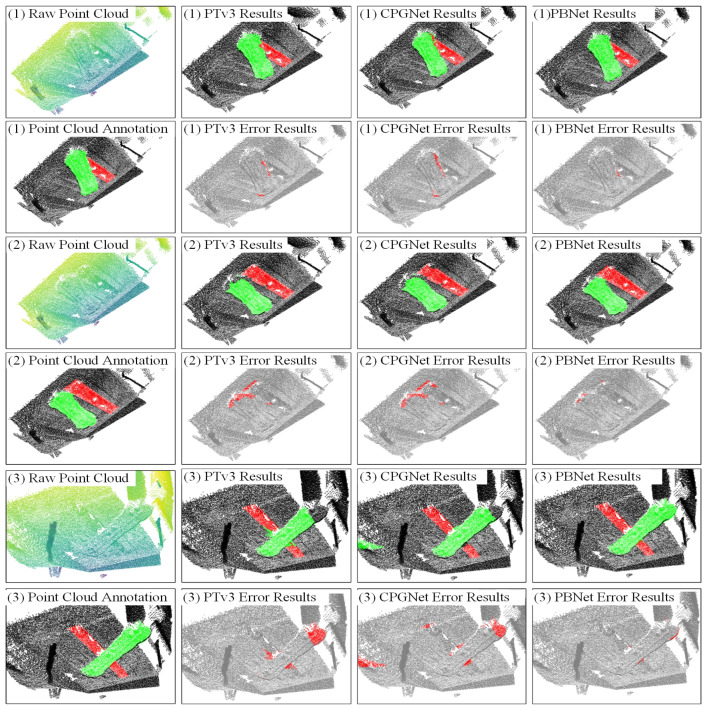
Qualitative comparison of semantic segmentation results on real-scene forging point clouds. The figure presents two sets of visual comparisons for point cloud segmentation. Labels (1)–(3) correspond to three different test samples, where (1), (2), and (3) denote the first, second, and third samples, respectively.In the result maps of each method, green points denote forgings, and red points denote cavities; other colors indicate background or non-target points.In the error maps, red points indicate misclassified points.

A qualitative evaluation was conducted on the fine-tuned models, and the results are presented in Figure 8. This figure illustrates three representative samples (labeled (1)–(3)) and compares the segmentation results of the proposed PBNet with those of the current state-of-the-art methods PTv3 [16] and CPGNet [11]. To intuitively highlight segmentation errors, error results are also provided, where red dots indicate regions where the predictions are inconsistent with the ground truth.

Specifically, in sample (1), PTv3 and CPGNet exhibit substantially more errors than PBNet, with the majority of incorrect regions concentrated at the interface between the forging workpiece and the die cavity, i.e., where the forging occludes the cavity, indicating that both methods have difficulty handling local occlusions. In sample (3), the forging and cavity deeply overlap, forming complex occlusions; PTv3 produces extensive misclassification of forging points, and CPGNet even incorrectly labels portions of the background as forging targets. In contrast, PBNet accurately separates the two and preserves clear and continuous segmentation boundaries at occlusion edges. In sample (2), although the forging and cavity have a small contact area and the overall structure remains intact, PTv3 and CPGNet still generate a large number of misclassified points along edges and fine structures, reflecting their sensitivity to geometric details and insufficient stability. PBNet, on the other hand, yields significantly fewer prediction errors and demonstrates stronger robustness and structural consistency in both complex boundary areas and regions with minimal interference.

Taken together, the qualitative analysis in Figure 8 shows that, compared with PTv3 and CPGNet, the proposed PBNet exhibits significant advantages when confronted with typical industrial challenges such as occlusions, background interference, and complex geometric structures. It achieves more robust and accurate semantic segmentation in real forging scenarios, thereby substantiating the reliability and superiority of the proposed method for practical applications.

### 4.4. Ablation Study

Impact of network modules. To assess the individual contributions of each module to the network’s overall performance, we conducted an ablation study. Specifically, the model was trained using the entire synthetic point-cloud semantic dataset along with 20% of the real-world forging point-cloud semantic data, while the remaining 80% of the real forged point clouds were reserved as a validation set. Based on this setup, we evaluated the following components separately: the star-based encoding module (SEM), the dual-branch downsampling module (DDM), the multi-level feature alignment module (MFAM), and the weighted feature fusion module (WFFM). The results are summarised in Table 3. The first row shows the full model with all modules included, achieving a best mIoU of 85.9%. In the second row, SEM was replaced with a standard ResBlock [40], while all other structures remained unchanged—mIoU dropped to 80.1%, a decrease of 5.8%. This indicates that SEM offers substantially superior feature extraction compared to a standard residual block. In the third row, DDM was substituted by traditional MaxPooling downsampling; the mIoU fell to 82.1%, a 3.8% drop relative to the full model, demonstrating that DDM more effectively preserves multi-scale contextual information. The fourth row removed the alignment mechanism of MFAM in the decoder, replacing it with simple feature concatenation; mIoU decreased to 81.3%, a reduction of 4.6%, confirming the importance of feature alignment in fusing multi-level information. In the fifth row, WFFM’s weighted fusion was replaced with simple concatenation, resulting in an mIoU of 83.2%, a 2.7% decline—indicating that weighted fusion more effectively selects and combines the most salient features.

In summary, all modules positively contribute to improving PBNet’s performance. Among them, SEM and MFAM have particularly significant impacts on overall performance, further validating the effectiveness and necessity of the module designs proposed herein.

Encoder Backbone Impact. To thoroughly evaluate the performance advantages of the proposed Star-Shaped Encoding Module (SEM), we conducted comparative experiments replacing SEM in PBNet with several current mainstream lightweight encoding modules. Specifically, we substituted SEM with MobileOne Block [41], ShuffleNet Block [42], MobileNetV3 Block [43], and Ghost Block [44], respectively, while keeping the rest of the network architecture and training configuration unchanged. The comparative results are presented in Table 4. Among all compared modules, SEM achieved uniformly superior performance. Its overall mIoU reached 85.9%, outperforming the second-best MobileOne Block by 2.6%, and markedly surpassing the ShuffleNet, MobileNetV3, and Ghost alternatives. For the specific sub-categories “Forging” and “Cavity”, SEM likewise maintained the highest accuracy, demonstrating its superior encoding capability in capturing the critical geometric features of forged-part point clouds. This comparative experiment clearly indicates that SEM significantly outperforms other mainstream lightweight designs in accuracy and is a key component ensuring PBNet’s high-precision segmentation.

Random Gaussian Noise Impact. As shown in Table 5, when the noise variance is set to $\delta^{2}=0.02$, the model achieves the highest mIoU on the test set (85.9%), representing a clear improvement over the noise-free baseline (84.3%). In contrast, excessively strong noise ($\delta^{2}>0.02$) leads to a degradation in performance, indicating that overly large perturbations can disrupt the effective structural information in the input data. Therefore, selecting $\delta^{2}=0.02$ as the noise intensity strikes a favorable balance between enhancing model robustness and preserving feature integrity, validating the rationality and effectiveness of this setting.

## 5. Conclusions

In this paper, we propose a novel semantic segmentation method tailored for the die-forging environment, PBNet, which effectively addresses the misclassification issues caused by drastic changes in workpiece pose and non-rigid surface deformations during the forging process. To tackle the challenges of complex geometric structures and significant domain shifts inherent to this scenario, PBNet incorporates three key architectural innovations: First, a Star-based Encoding Module (SEM) is introduced in the Bird’s-Eye-View (BEV) branch, significantly enhancing local-to-global feature modeling in BEV space; compared with the MobileOne backbone, this yields a 2.6% improvement in mIoU. Second, during decoding, a Multi-level Feature Alignment Module (MFAM) is designed to effectively mitigate spatial misalignment among multi-scale features caused by deformation. Finally, at the fusion stage, a Weighted Feature Fusion Module (WFFM) is proposed to achieve adaptive fusion of point cloud geometric features and BEV semantic representations. We conduct a comprehensive evaluation on our self-constructed simulated and real die-forging point cloud datasets. Experiments demonstrate that PBNet, trained solely on simulated data and directly tested in real scenarios, achieves an mIoU approximately 2% higher than the state-of-the-art PointTransformer v3 (PTv3). With minor fine-tuning using a small amount of real data, performance improves further, outperforming all compared methods. These results fully validate the robustness and generalization ability of PBNet in industrial scenarios with strong deformations and cross-domain challenges, providing support for precise 3D semantic understanding in die-forging environments.

## Figures and Tables

**Table 1:** Test results on the real-world forging dataset after training on the simulated forging dataset.

Methods	Publication	Forging (%)	Cavity (%)	Speed (ms)	MIoU (%)
KPConv [15]	2019	71.8	71.0	−	71.4
RandLA-Net [4]	2020	74.2	73.1	420	73.7
PointNext [37]	2022	75.3	74.2	−	74.8
OctFormer [38]	2023	77.4	76.3	91	76.9
PTv3 [16]	2024	79.1	78.6	70	78.85
SparseConvNet [39]	2018	70.1	69.5	200	69.8
SPVNAS [5]	2020	78.2	77.9	160	78.0
Cylinder3D [6]	2020	79.5	78.4	170	78.9
RPVNet [10]	2021	80.1	79.4	165	79.8
CPGNet [11]	2022	79.5	78.9	50	79.2
PBNet (Ours)	-	81.6	80.3	30	80.9

**Table 2:** Evaluation results after fine-tuning using partial real-world data.

Methods	Publication	Forging (%)	Cavity (%)	Speed (ms)	MIoU (%)
RandLA-Net [4]	2020	78.4	77.6	420	78.0
OctFormer [38]	2023	82.6	80.9	91	81.8
PTv3 [16]	2024	85.2	83.6	70	84.4
SparseConvNet [39]	2018	74.3	72.1	200	73.2
SPVNAS [5]	2020	83.6	82.9	160	83.25
Cylinder3D [6]	2020	84.7	83.5	170	84.1
RPVNet [10]	2021	85.1	84.4	165	84.8
CPGNet [11]	2022	84.5	83.9	50	84.2
PBNet (Ours)	-	86.6	85.3	30	85.9

**Table 3:** Impact of each network module.

NO.	SEM	DDM	MFAM	WFFM	mIoU
1	**✓**	**✓**	**✓**	**✓**	85.9
2	**✗**	**✓**	**✓**	**✓**	80.1
3	**✓**	**✗**	**✓**	**✓**	82.1
4	**✓**	**✓**	**✗**	**✓**	81.3
5	**✓**	**✓**	**✓**	**✗**	83.2

*Note:* A checkmark (**✓**) indicates the presence of the corresponding module.

**Table 4:** Impact of Star-Based Encoding Module (SEM).

Module	Forging $\left(\%\right)$	Cavity $\left(\%\right)$	mIoU $\left(\%\right)$
MobileOne Block [41]	83.8	82.7	83.3
ShuffleNet Block [42]	81.8	80.1	80.9
MobilenetV3 Block [43]	80.2	79.1	79.7
Ghost Block [44]	79.4	78.7	79.1
SEM	86.6	85.3	85.9

**Table 5:** The Effect of Random Gaussian Noise $N\left(\mu =0,\delta^{2}\right)$.

$\delta^{2}$	0.0	0.01	0.02	0.03	0.04	0.05
**mIoU $\left(\%\right)$**	84.3	84.6	85.9	85.5	85.0	84.7

## Data Availability

All relevant data are available from the corresponding author upon request.

## Appendix A

The encoder for the BEV branch comprises star-based encoding modules, as illustrated in Figure 4. In a single-layer neural network, the star operation is generally formulated as $\left(W_{1}^{T}X+B_{1}\right)*\left(W_{2}^{T}X+B_{2}\right)$, which integrates features from two distinct linear transformations via element-wise multiplication. To simplify notation, the weight matrix and bias term can be combined into a single entity denoted as $\hat{W}=\left[W,B\right]$, while the input is extended as $\hat{X}=\left[X,1\right]$. This allows the star operation to be rewritten as $\left({\hat{W}}_{1}^{T}\hat{X}\right)*\left({\hat{W}}_{2}^{T}\hat{X}\right)$. For analytical convenience, this paper focuses on the scenario involving a single output channel and a single input element, specifically with $w_{1},w_{2},x\in R^{(c_{d}+1)\times 1}$, where $c_{d}$ is the number of input channels. The framework can be readily extended to multiple output channels, i.e., ${\hat{W}}_{1},{\hat{W}}_{2}\in R^{\left(c_{d}+1\right)\times \left(c_{d}^{\prime }+1\right)}$, and can also handle multiple input feature elements, in which case $\hat{X}\in R^{(c_{d}+1)\times n}$. In summary, the general form of the star operation [34] can be uniformly expressed as follows:

(A1) $$\begin{array}{cc} \; & w_{1}^{T}x*w_{2}^{T}x=\underset{\left(c_{d}+2\right)\left(c_{d}+1\right)/2\;\mathrm{items}\;}{\underbrace{\alpha_{\left(1,1\right)}x^{1}x^{1}+\cdots +\alpha_{\left(4,5\right)}x^{4}x^{5}+\cdots +\alpha_{\left(c_{d}+1,c_{d}+1\right)}x^{c_{d}+1}x^{c_{d}+1}}} \end{array}$$

(A2) $$\begin{array}{c} \alpha_{\left(i,j\right)}=\left\{\begin{array}{cc} w_{1}^{i}w_{2}^{j} & \;\mathrm{if}\;i==j,\\ w_{1}^{i}w_{2}^{j}+w_{1}^{j}w_{2}^{i} & \;\mathrm{if}\;i!=j. \end{array}\right. \end{array}$$

In Equation (A1), $x_{k}$ denotes the *k*-th component of the input feature vector $x={\left(x^{1},x^{2},\ldots ,x^{c_{d}+1}\right)}^{⊤}$. In Equation (A2), the indices *i* and *j* are used to traverse the channels of the weight matrices $w_{1}$ and $w_{2}$, while α denotes a per-element coefficient, where *i* and *j* to index the channels, and α denotes a per-element coefficient.

After reformulating the star-based operation described in Equation (A1), we can extend it into a combination of $\frac{\left(c_{d}+2\right)\left(c_{d}+1\right)}{2}$ distinct terms. It is worth noting that, except for the term $\alpha_{\left(c_{d}+1,c_{d}+1\right)}x^{c_{d}+1}x^{c_{d}+1}$, all other terms exhibit a nonlinear relationship with the input x, which suggests they correspond to several independent implicit dimensions. Although we employ a computationally efficient star-based operation in the *d*-dimensional space, it effectively achieves feature representation in a higher-dimensional implicit feature space $\frac{\left(c_{d}+2\right)\left(c_{d}+1\right)}{2}\approx {\left(\frac{c_{d}}{\sqrt{2}}\right)}^{2}$ (where $c_{d}\gg 2$). This significantly expands the feature dimensionality without introducing any additional computational overhead in a single-layer structure. Assuming the initial network layer has a width of $c_{d}$, applying a single star-based operation yields the result shown in Expression (A1), mapping the features into a representation within the implicit feature space $R^{{\left(\frac{c_{d}}{\sqrt{2}}\right)}^{2^{1}}}$. Furthermore, stacking multiple such layers in a recursive manner leads to an exponential growth of the implicit dimensionality, ultimately approaching infinity. Let $O_{l}$ denote the output of the *l*-th star-based operation; it can be expressed as follows:

(A3) $$\begin{array}{c} O_{l}=W_{l,1}^{T}O_{l-1}*W_{l,2}^{T}O_{l-1}\in R^{{\left(\frac{c_{d}}{\sqrt{2}}\right)}^{2^{l}}} \end{array}$$

In other words, by stacking *l* layers of the star-shaped operation, we can implicitly obtain an extremely high-dimensional representation in the feature space R. For example, given a 10-layer isotropic network with a width of 128 per layer, the implicit feature dimension achieved through the star-shaped operation can reach approximately $90^{1024}$ dimensions, which is already close to being considered infinite-dimensional. Therefore, even with only a few stacked layers, the star-shaped operation is capable of expanding the implicit feature dimension exponentially.
